# P-gp activity is a critical resistance factor against AVE9633 and DM4 cytotoxicity in leukaemia cell lines, but not a major mechanism of chemoresistance in cells from acute myeloid leukaemia patients

**DOI:** 10.1186/1471-2407-9-199

**Published:** 2009-06-23

**Authors:** Ruoping Tang, Simy Cohen, Jean-Yves Perrot, Anne-Marie Faussat, Claudia Zuany-Amorim, Zora Marjanovic, Hamid Morjani, Fanny Fava, Elise Corre, Ollivier Legrand, Jean-Pierre Marie

**Affiliations:** 1 Assistance Publique – Hôpitaux de Paris, Hôpital Hôtel Dieu, 1 place du Parvis de Notre-Dame, 75181 Paris cedex 04, France; 2 Université Pierre et Marie Curie, UMRs 872, Equipe 18, 1 place du Parvis de Notre-Dame, 75181 Paris cedex 04, France; INSERM, 1 place du Parvis de Notre-Dame, 75181 Paris cedex 04, France; 3 SANOFI-AVENTIS, Vitry-sur-Seine, France; 4 JE Onco-Pharmacologie, IFR53; UFR de Pharmacie, 51096 Reims cedex, France

## Abstract

**Background:**

AVE9633 is a new immunoconjugate comprising a humanized monoclonal antibody, anti-CD33 antigen, linked through a disulfide bond to the maytansine derivative DM4, a cytotoxic agent and potent tubulin inhibitor. It is undergoing a phase I clinical trial. Chemoresistance to anti-mitotic agents has been shown to be related, in part, to overexpression of ABC proteins. The aim of the present study was to investigate the potential roles of P-gp, MRP1 and BCRP in cytotoxicity in AVE9633-induced acute myeloid leukaemia (AML).

**Methods:**

This study used AML cell lines expressing different levels of P-gp, MRP1 or BCRP proteins and twenty-five samples from AML patients. Expression and functionality of the transporter protein were analyzed by flow cytometry. The cytotoxicity of the drug was evaluated by MTT and apoptosis assays.

**Results:**

P-gp activity, but not MRP1 and BCRP, attenuated AVE9633 and DM4 cytotoxicity in myeloid cell lines. Zosuquidar, a potent specific P-gp inhibitor, restored the sensitivity of cells expressing P-gp to both AVE9633 and DM4. However, the data from AML patients show that 10/25 samples of AML cells (40%) were resistant to AVE9633 or DM4 (IC_50 _> 500 nM), and this was not related to P-gp activity (p-Value: 0.7). Zosuquidar also failed to re-establish drug sensitivity. Furthermore, this resistance was not correlated with CD33 expression (p-Value: 0.6) in those cells.

**Conclusion:**

P-gp activity is not a crucial mechanism of chemoresistance to AVE9633. For patients whose resistance to conventional anthracycline AML regimens is related to ABC protein expression, a combination with AVE9633 could be beneficial. Other mechanisms such as microtubule alteration could play an important role in chemoresistance to AVE9633.

## Background

Acute myeloid leukaemia (AML) is characterised by the proliferation of clonal precursor myeloid cells with arrested differentiation and subsequent accumulation of myeloid blasts in the bone marrow. Approximately 60%–80% of younger adults with AML achieve complete remission (CR) with conventional chemotherapy such as cytarabine and an anthracycline. However, a significant proportion of the responsive patients suffer relapses and die of treatment-refractory disease. The treatment of relapsed AML patients is considerably less successful, especially in the elderly, because the toxicity of standard induction chemotherapy is poorly tolerated in the older age group [1,2]. Thus, novel drugs and treatment strategies are major objectives of research; conjugates of antibodies with powerful cytotoxic agents have been explored. Gemtuzumab ozogamicin (GO) is the first immunoconjugate approved by the United States Food and Drug Administration (FDA) for treating refractory AML [3]. Sanofi Aventis and ImmunoGen have developed a novel immunoconjugate, AVE9633, which has been evaluated in Phase I clinical trials on refractory AML patients.

AVE9633 is an antibody-drug conjugate comprising the cytotoxic maytansinoid drug DM4 (N^2'^-deacetyl-N^2'^-(4-methyl-4(oxobutyldithio)-1-oxopentyl)-maytansine) linked via disulfide bonds to the anti-CD33 monoclonal antibody huMy9-6. CD33 is a transmembrane cell surface glycoprotein receptor that is specific for myeloid cells. Its expression is regulated during maturation of the myeloid lineage, resulting in low level expression on peripheral granulocytes and tissue macrophages [4]. The CD33 antigen is expressed on approximately 90% of AML myeloblasts, including leukaemic clonogenic precursors as well as normal myeloid precursor cells, but not on CD34^+ ^pluripotent hematopoietic stem cells or in non-haematopoietic tissues [5]. It represents an attractive target for antibody-based therapy in patients with AML. The immunoconjugate AVE9633 binds target cells expressing CD33 and is subsequently internalised. The DM4 is released within the cell and exerts its cytotoxic activity. The Phase I clinical trial [6] has provided evidence that AVE9633 has anti-leukaemia activity and may be given as an outpatient treatment.

DM4, a structural analogue of maytansine, is a new thiol-containing and potent maytansinoid. It was synthesized in order to link maytansinoids to antibodies via disulfide bonds. Maytansinoids inhibit tubulin polymerization and microtubule assembly and enhance microtubule destabilization, so there is potent suppression of microtubule dynamics resulting in a mitotic block and subsequent apoptotic cell death [7]. They are approximately 200–1000 times as active as the Vinca alkaloids. Maytansine was evaluated by the National Cancer Institute in Phase I and II studies during the 1970s [8-12]. Although complete and partial regressions of several cancers such as lymphoma, melanoma and acute lymphocytic leukaemia were noted, severe toxic effects were observed in those early clinical trials.

Maytansine is a natural product, originally derived from the Ethiopian shrub *Maytansine serrata*, so it may be a substrate of ABC proteins. One of the best-characterized mechanisms of chemoresistance in AML is P-glycoprotein (P-gp or ABCB1) expression; P-gp serves as an energy-dependent efflux pump that extrudes chemotherapeutic agents out of cells [13]. Its expression is particularly high in older adults and in those with relapsed and refractory AML and is associated with poor prognosis [14,15]. Other ABC (ATP-binding-cassette) proteins such as ABCC1 (MRP1), ABCC3 (MRP3) and ABCG2 (BCRP) are also associated with a poor outcome [16]. Compelling data from the literature demonstrate that expression of P-gp and MRP1 attenuates the *in vitro *cytotoxic activity of the GO immunoconjugate in AML cells. In addition, inhibition of P-gp and MRP1 function by CsA, Zosuquidar or MK-571 restores GO sensitivity [17,18]. It has been demonstrated that P-gp activity *in vivo *is associated with a worse prognosis in the clinical response to GO [19].

In this study, we investigated the potential role of P-gp, MRP1 and BCRP in modulating AVE9633 and DM4 cytotoxicity using cell lines specifically expressing those proteins, and also using cells from AML patients. We also investigated whether Zosuquidar modulates AVE9633 and DM4 cytotoxicity and studied its relationship to P-gp activity and CD33 expression.

## Methods

### Cell lines and cell culture

Eight cell lines expressing different levels of three ABC proteins, P-gp, MRP1 and BCRP, were studied. One series of cell lines was K562 (human bcr-abl myeloid leukaemia cells) and its derivatives: K562/HHT40 and K562/HHT90 (developed in our laboratory) [20], and K562/Dox and K562/BCRP [21] (kindly provided by Y. Sugimoto, Foundation for Cancer Research in Japan). The other cell lines were HL60, a human myeloid leukaemia cell line, and its derivatives: HL60/DNR and HL60/ADR (gifted by F. Lacombe, Bordeaux, France and F. Calvo, Hôpital Saint-Louis, Paris, France, respectively), which were developed respectively as Daunorubicin- and Adryamicin-resistant lines. All the cell lines were cultured in RPMI-1640 medium supplemented with 10% foetal bovine serum, 2 mM glutamine, 50 U/ml penicillin, 50 μg/ml streptomycin, and were incubated at 37°C in a humidified atmosphere containing 5% CO_2_.

### AML patient samples

Peripheral blood samples from 25 AML patients were obtained from cell bank ("Tumorothèque Leucémies" Hôtel-Dieu N°579). Mononuclear cells (MNC) were isolated using Ficoll-Hypaque density gradients. The fresh leukaemia cells were cultured under the same general conditions as the cell lines.

### Ethical approval

The present study carried out on human blood cells is in compliance with the Helsinki Declaration, and was approved by the French Institute National of Cancer ("Tumorothèque Leucémies" Hôtel-Dieu N°579). AML patient blood samples were obtained after their informed consent (Formulary EORTC study N°06012).

### Drugs and modulators

AVE9633 and DM4 were kindly provided by the Sanofi-Aventis Research Division (France).

Modulators of ABC proteins were Zosuquidar for P-gp (Kanisa, USA), MK-571 for MRP1 and Fumitrimorgin C (FTC) for BCRP (Alexis Biochemicals, USA).

### P-gp, MRP and BCRP expression

To detect the extracellular P-gp epitopes, cells (1 × 10^6^) were incubated for 30 min with phycoerythrin (PE)-conjugated UIC2 (Immunotech, France) or phycoerythrin-conjugated isotype IgG2a as control. To detect the intracellular epitopes of MRP1 and BCRP, cells (1 × 10^6^) were first fixed and permeabilized using IntrPrep™ (Beckman Coulter, Villepinte, France) according to the manufacturer's instructions, then incubated with the primary antibodies MRPm6 (MRP1, Monosan, Netherlands) or Bxp21 (BCRP, Immunotech, France) or an isotype control antibody (IgG1) for 30 min at room temperature. After one wash, the cells were incubated with a secondary phycoerythrin-conjugated goat anti-mouse IgG (H+L). The fluorescence intensity was measured using a flow cytometer (EPICS Altra, Beckman Coulter). ABC protein expression was determined by the ratio of the mean fluorescence intensity of each specific antibody to the control antibody (P-gp/IgG2a; MRP1/IgG1, BCRP/IgG1). For each sample, 5,000 events were collected. All the experiments were performed in triplicate.

### P-gp, MRP and BCRP activity

P-gp, MRP and BCRP activities were evaluated by the uptake of their specific fluorescent substrates in the presence or absence of their specific modulators. Cells (1 × 10^6^) were incubated respectively with DiOC_2_(3) (25 nM) ± Zosuquidar (0.3 μM) for P-gp, Calcein-AM (C-AM) (0.2 μM) ± MK571 (5 μM) for MRP or mitoxantrone (3 μM) ± Fumitremorgin C (FTC) (10 μM) for BCRP for 30 min at 37°C. The samples were then analysed by flow cytometry. Dye uptake was expressed as a D value ranging from 0 (no difference) to 1 (no overlap) generated by a Kolmogorov-Smirnov test, which was used to determine the distribution of the mean fluorescence intensity (MFI) between the presence and absence of each modulator. For each sample, 5,000 events were collected. All the experiments were performed in triplicate.

### Cell viability (MTT assay)

Cells (2 × 10^4^/well for cell lines, 4 × 10^5^/well for patient cells) were cultured in 96-well plates. Different concentrations of compounds were added in the presence or absence of Zosuquidar (0.3 μM), MK571 (5 μM) or FTC (1 μM). After three days' incubation for DM4 and four for AVE9633, 20 μl of MTT (5 mg/ml, 3-(4,5-dimethyl-2-thiazolyl)-2,5-diphenyl-2H-tetrazolium bromide) was added to each well for a further 4 h incubation. The purple precipitate was dissolved in 200 μl DMSO and the optical density was measured using a multiwell plate reader (Multiskan Ascent, Labsystems). Each condition was repeated in four wells, and the results are expressed as the means of the four wells. Viability is expressed as the ratio of the optical density of the cells in the presence of each drug at different concentrations with or without modulator and the control cells in the medium alone. The concentration effecting 50% inhibition (IC_50_) was determined by CalcuSyn Software (Biosoft, Cambridge, UK) following the viability results. All the experiments were performed in triplicate.

### Apoptosis analysis

Cells (10^6^) were stained with AnnexinV-FITC and propidium iodide (PI) in calcium-HEPES buffer for 15 min, as instructed by the manufacturer (Roche, France). The percentage of apoptotic cells was determined by flow cytometry.

### Statistical analysis

Statistical discovery software (JMP5.1) was employed, using Student's t-test for each pair comparison.

## Results

### P-gp, MRP1 and BCRP expression and activity in human leukaemia cell lines

First, we examined the expression and activity of P-gp, MRP1 and BCRP in the cell lines we used, HL60 and K562 (sensitive) and their variants (resistant). The expression and activity results are displayed in Table 1. The P-gp, MRP1 and BCRP protein expression results agreed well with their activities (r = 0.85, logarithmic). There was no expression or activity of any of those ABC proteins in the parental HL60 and K562 cells. However, the resistant cell lines, K562/HHT40, K562/HHT90 and K562/DOX, showed marked P-gp expression and activity at different levels, while MRP1 and BCRP expression and activity were comparable to those of the parental cell lines. P-gp expression and activity were increased in those cell lines (Table 1) and was also very high in HL60/DNR cells (Table 1). HL60/ADR cells showed increased MRP1 expression and MRP activity, but no expression or activity of the other proteins (Table 1). K562/BCRP cells specifically showed BCRP expression and activity, but not the other proteins (Table 1). It is important to note that no cross-resistance was observed in these cell lines.

**Table 1:** P-gp, MRP, BCRP expression and activity in K562, HL60 and their variant cell

	Expression			Activity		
	**P-gp**	**MRP1**	**BCRP**	**P-gp**	**MRP**	**BCRP**
	UIC2	MRPm6	BXP21	DiOC_2 _(3)/zosuquidar	C-AM/MK571	Mitox/FTC
HL60	1.14 ± 0.27	1.2 ± 0.19	1.54 ± 0.79	0	0.09 ± 0.08	0
HL60/DNR	19.30 ± 4.79	0.91 ± 0.52	1.47 ± 0.61	0.98 ± 0.004	0.09 ± 0.04	0
HL60/ADR	1.50 ± 0.44	3.15 ± 0.07	1.56 ± 0.77	0	0.91 ± 0.04	0.17 ± 0.13
K562	0.98 ± 0.17	1.35 ± 0.37	1.25 ± 1.04	0	0.03 ± 0.06	0.043 ± 0.06
K562/HHT40	2.48 ± 0.60	1.53 ± 0.52	1.52 ± 0.51	0.41 ± 0.01	0.17 ± 0.05	0.13 ± 0.14
K562/HHT90	3.24 ± 0.80	1.2 ± 009	1.65 ± 0.64	0.83 ± 0.05	0.22 ± 0.14	0.05 ± 0.07
K562/Dox	11.58 ± 3.42	1.43 ± 0.37	1.85 ± 0.46	0.99 ± 0.01	0.13 ± 0.09	0
K562/BCRP	1.17 ± 0.2	1.13 ± 0.30	7.57 ± 0.54	0	0	0.59 ± 0.11

### Effect of P-gp activity and its modulator Zosuquidar on DM4 and AVE9633 cytotoxicity

To determine the potential role of P-gp protein in DM4 and AVE9633 cytotoxicity, sensitivity to DM4 and AVE9633 was first examined in the parental cell line HL60 and its variant that specifically expressed P-gp. K562 and its variant cell lines were only used to test the cytotoxicity of DM4 because they do not express CD33 antigen. The IC_50 _values of DM4 and AVE9633 in those cell lines were assessed by MTT assays. As illustrated in Table 2, the IC_50 _values of DM4 in K562/HHT40 (18.4 nM ± 1.2), K562/HHT90 (36.6 nM ± 2.0), K562/DOX (437.6 nM ± 15.1) and HL60/DNR (>800 nM) were higher than in the parental K562 (11.8 nM ± 1.1) and HL60 (14.8 nM ± 0.3) cells. The increase depended on the activity of P-gp: the more active the P-gp, the higher the IC_50 _of DM4 (r = 0.70, logarithmic).

**Table 2:** The cytotoxicity of DM4 and AVE9633 in K562, HL60 and their variant cell lines expressing P-gp in the presence or absence of the P-gp modulator Zosuquidar

IC_50_	HL60	HL60/DNR	K562	K56/HHT40	K562/HHT90	K562/DOX
AVE9633 (nM)	19.9 ± 0.7	> 800				
AVE9633 + Zosuquidar	20.1 ± 1.1	10.4 ± 2.1				
						
DM4 (nM)	14.8 ± 0.3	> 800	11.8 ± 1.1	18.4 ± 1.2	36.6 ± 2.0	437.6 ± 15.1
DM4 + Zosuquidar	18.3 ± 1.3	10.9 ± 0.1	12.6 ± 1.2	6.5 ± 0.6	10.1 ± 0.8	11.6 ± 0.9

* Zosuquidar: 0.3 μM

The IC_50 _of AVE9633 in HL60/DNR cells was also above 800 nM, far higher than in the parental HL60 cells (19.9 nM ± 0.7).

We also examined whether Zosuquidar, a specific P-gp inhibitor, restored the sensitivity to DM4 and AVE9633 in cells with active P-gp. In the presence of Zosuquidar, the IC_50 _values of DM4 in K562/HHT40, K562/HHT90, K562/DOX and HL60/DNR (Table 2) were respectively 6.5 ± 0.6, 10.1 ± 0.8, 11.6 ± 0.9 and 10.9 ± 0.1 nM, similar to or less than those in the parental K562 (11.8 ± 1.1) and HL60 (14.8 ± 0.3) cells. Similar results were also observed for HL60/DNR with AVE9633 in the presence of Zosuquidar. The IC_50 _of AVE9633 in HL60/DNR cells was >800 nM without Zosuquidar versus 10.4 ± 2.1 nM with Zosuquidar, close to the value observed in HL60 cells.

**Figure 1:**
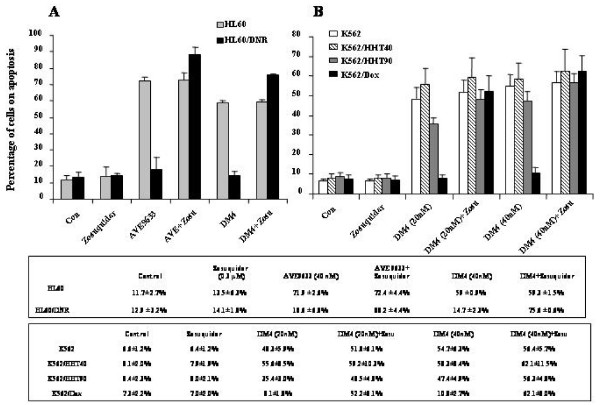
**Apoptosis induced by DM4 and/or AVE9633 in presence or absence of Zosuquidar in HL60, K562 and their variant P-gp expressing cells**. Cells were treated with DM4 or AVE9633 at 20 nM or/and 40 nM in the presence or absence of Zosuquidar (0.3 μM) for 48 h, then stained with Annexin V/propidium iodide for flow cytometry. All experiments were done in triplicate. A: HL60 (grey) and HL60/DNR (black). B: K562 (blank), K562/HHT40 (stripe), K562/HHT90 (grey) and K562/Dox (black).

To confirm that the cytotoxicity of AVE9633 and DM4 was modulated by overexpression of P-gp, we examined the induction of apoptosis by these agents in the presence or absence of Zosuquidar in cells with active P-gp. HL60, HL60/DNR, K562, K562/HHT40, K562/HHT90 and K562/Dox cells were treated for 48 or 72 h with DM4 or AVE9633 alone or in the presence of Zosuquidar, then stained with Annexin V/PI and analysed by flow cytometry. As shown in figure 1, DM4 and AVE9633 alone at 40 nM induced a marked apoptotic response in HL60 cells (DM4: 59 ± 0.9% and AVE9633: 71.9 ± 2.6%) but not in the P-gp-functional HL60/DNR cells (DM4:14.7 ± 2.3% and AVE9633: 18.6 ± 6.8%). However, in the presence of the P-gp inhibitor Zosuquidar, DM4 and AVE9633 triggered apoptosis significantly in the HL60/DNR cells, inducing 75.6 ± 0.6% (DM4) and 88.2 ± 4.4% (AVE9633) cell death.

The detailed results for K562, K562/HHT40, K562/HHT90 and K562/Dox are shown in figure 1. We tested their sensitivity to two concentrations of DM4, 20 nM and 40 nM. The lower concentration (20 nM) is greater than the IC_50 _of DM4 in K562/HHT40 cells by the MTT test, but lower than that in K562/HHT90 cells; 40 nM is greater than the IC_50 _in K562/HHT90. We observed that DM4 at both 20 nM and 40 nM with or without Zosuquidar induced apoptosis in K562 and K562/HHT40 cells with similar efficacy, but fewer apoptotic cells were induced in K562/HHT90 by DM4 at 20 nM (35.4 ± 3.0%), but similar at 40 nM (47.4 ± 4.9%); this resistance to DM4 at 20 nM was restored by Zosuquidar (48.5 ± 4.8%). DM4 alone at 20 nM and 40 nM did not induce apoptosis in K562/Dox cells, only 8.1 ± 1.8% and 10.8 ± 2.7% respectively, but in the presence of Zosuquidar the apoptosis rates were 52.2 ± 8.1% and 62.1 ± 8.0% respectively.

### Effect of MRP activity and its modulator MK571 on DM4 and AVE9633 cytotoxicity

**Figure 2:**
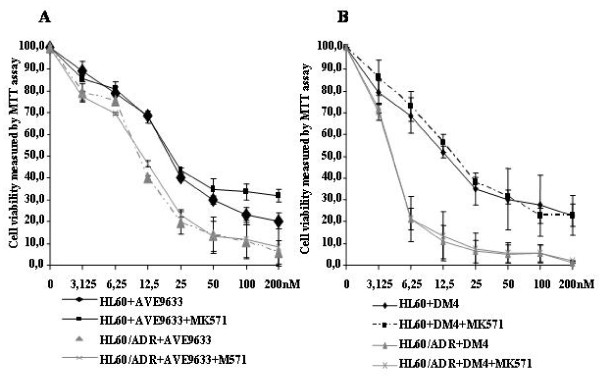
**The sensitivity of HL60 and HL60/ADR cells to DM4 and AVE9633 with or without MRP inhibitor**. HL60 and HL60/ADR cells were treated with AVE9633 (A) or DM4 (B) at different concentrations (3.125, 6.25, 12.5, 25, 50, 100 and 200 nM) in the presence or absence of MK571 (5 μM) for 72 h, then their viability was measured by an MTT assay. All experiments were done in triplicate.

**Figure 3:**
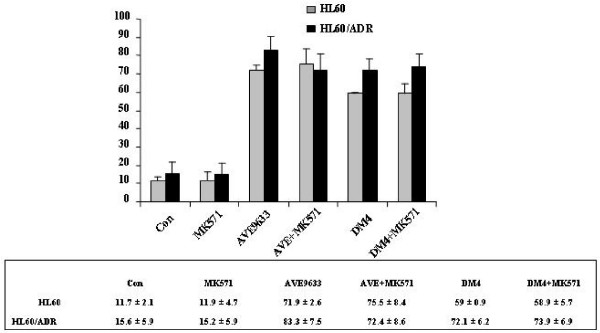
**Apoptosis induced by DM4 and AVE9633 in the presence or absence of MRP inhibitor MK571 in HL60 an HL60/ADR cells**. HL60 (grey) and HL60/ADR (black) cells were treated with DM4 or AVE9633 at 40 nM in the presence or absence of MK571 (5 μM) for 48 h, then stained with Annexin V/propidium iodide for flow cytometry. All experiments were done in triplicate.

To check the effect of MRP activity on DM4 and AVE9633 cytotoxicity, the viability and apoptosis of HL60 and its variant MRP^+ ^HL60/ADR cells treated with DM4 or AVE9633 alone and with MK571 were examined. HL60/ADR cells were more sensitive to DM4 and AVE9633 than the parental HL60 cells (Figure 2). The IC_50 _values of DM4 and AVE9633 in HL60/ADR were even lower than in the parental cells: 4.3 ± 0.3 and 10.5 ± 0.1 nM for HL60/ADR versus 16.3 ± 1.9 and 19.9 ± 0.7 for HL60. Addition of the MRP inhibitor (MK571) to those cells did not change their sensitivity to DM4 or AVE9633. We next investigated the induction of apoptosis in HL60 and HL60/ADR cells by 40 nM DM4 or AVE9633 alone or in the presence of MK571. The results also showed that the sensitivity of HL60/ADR cells to DM4 and AVE9633 was similar to that of the parental HL60 cells (Figure 3). Furthermore, MK571 (5 μM) failed to enhance the induction of apoptosis by DM4 or AVE9633 in HL60/ADR cells: 72.1 ± 6.2% or 83.3% ± 7.5 without MK571 versus 73.9 ± 6.9% or 72.4 ± 8.6% with MK571.

### Effect of BCRP activity and its modulator FTC on DM4 and AVE9633 cytotoxicity

**Figure 4:**
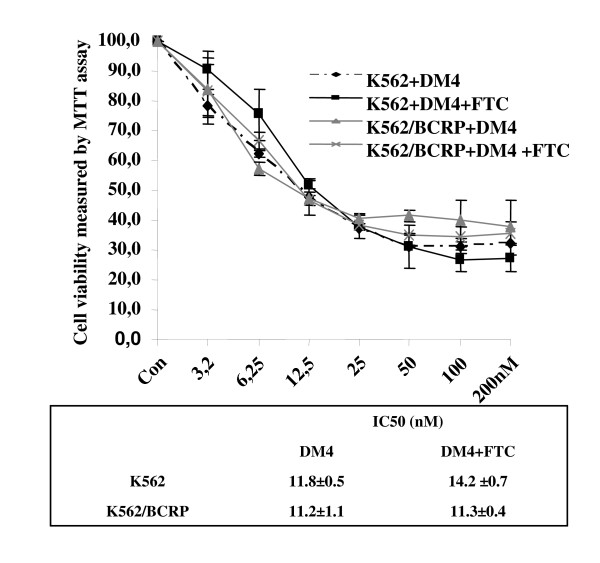
**The sensitivity of K562 and K562/BCRP cells to DM4 with or without BCRP inhibitor**. K562 and K562/BCRP cells were treated with DM4 at different concentrations (3.125, 6.25, 12.5, 25, 50, 100 and 200 nM) in the presence or absence of FTC (1 μM) for 72 h, and then their sensitivity was measured by an MTT assay. All experiments were done in triplicate.

The effect of BCRP on DM4 cytotoxicity was examined in K562 and K562/BCRP cells. The viability of K562/BCRP cells in the presence of DM4 was similar to that of the parental K562 cells (Figure 4). The BCRP inhibitor (FTC) did not change the response of those cells to DM4 significantly (Figure 4). The IC_50 _values of DM4 alone for K562 and K562/BCRP were respectively 11.8 ± 0.5 and 11.2 ± 1.1, versus 14.2 ± 0.7 and 11.3 ± 0.4 with FTC.

**Figure 5:**
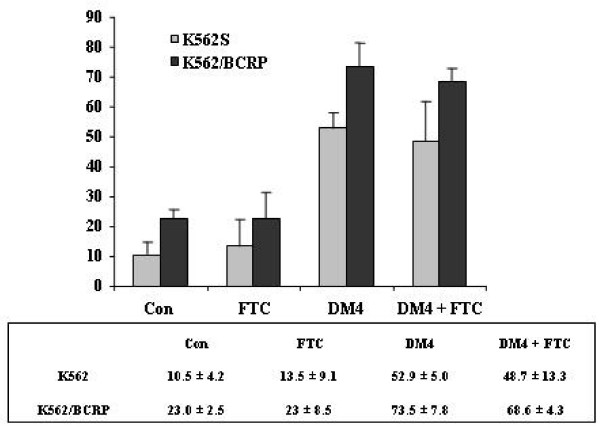
**Apoptosis induced by DM4 in the presence or absence of BCRP inhibitor FTC in K562 and K562/BCRP cells**. K562 (grey) and K562/BCRP (black) cells were treated with DM4 at 40 nM in the presence or absence of FTC (1 μM) for 48 h, and then stained with Annexin V/propidium iodide for flow cytometry. All experiments were done in triplicate.

The apoptosis induced in K562/BCRP cells by 40 nM DM4 alone or in the presence of FTC was comparable to that of the parental K562 cells (Figure 5). Also, FTC (1 μM) failed to enhance the induction of apoptosis by DM4 in K562/BCRP cells: 73.5 ± 7.8% without FTC versus 68.6 ± 4.3% with FTC.

### Sensitivity of AML patient cells to AVE9633 and DM4

We next tested the response of fresh cells from AML patients to DM4 and AVE9633 in order to confirm the effect of P-gp expression on AVE9633 and DM4 sensitivity, and to determine whether AVE9633 sensitivity was associated with CD33 expression in these cells. Thus, the sensitivity to DM4 and AVE9633, the expression of CD33 and the activity of P-gp were analysed in cells from 25 AML patients. As shown in Additional file 1, cells from 10 (40%) of the patients were highly resistant to AVE9633 or/and DM4; the IC_50 _of AVE9633 or DM4 was above 500 nM, the maximum concentration used *in vitro*, or above 200 nM for one patient's cells (there was a lack of concentrated AVE9633). At 200 nM, the viability of this patient's cells was about 95%. AVE9633-resistant cells from the patients were also resistant to DM4. Among the 10 non-responding patients, only three (12%) had cells with weak P-gp activity (P8, P12 and P17; D = 0.21, 0.24 and 0.26). Furthermore, pre-incubation of the cells with Zosuquidar did not restore the cytotoxicity of either AVE9633 or DM4. Interestingly, among the 15 responders (60%), the sensitivity to AVE9633 or DM4 in cells from three patients (12%) was enhanced by Zosuquidar (see Additional file 1); the cells from all three of these patients showed active P-gp (P16, P24 and P6; D = 0.25, 0.27 and 0.38). This result demonstrates that P-gp activity is not associated with AVE9633 and/or DM4 resistance (p-value: 0.7), and is not crucial for such resistance. This resistance to AVE9633 *in vitro *was not associated with CD33 expression in any patient (p-value: 0.6), nor was the sensitivity to AVE9633 among the responder patient cells.

We also compared the activity of AVE9633 to that of GO in 21 of the 25 patients in the presence or absence of Zosuquidar (see Additional file 1). Among 10 patients who were highly resistant to AVE9633 or/and DM4, the cells from eight were examined for GO response: the cells from four were sensitive to GO and those from the other four were insensitive. Among 15 patients who were sensitive to AVE9633 or/and DM4, the cells from 13 were examined for GO response; those from 10 were sensitive and those from the other three were resistant. Zosuquidar enhanced the cytotoxicity of GO in P-gp active cells from P16 and P6. This effect was more marked for GO than for AVE9633. However, Zosuquidar did not change the resistance status of P9.

## Discussion

P-gp, MRP1, MRP3 and BCRP activities have been shown to contribute to resistance to conventional cytarabine and anthracycline-based chemotherapy such as Daunorubicin, Idarubicin and Mitoxantrone in AML [13]. P-gp and MRP1 have also been associated with attenuated GO-induced cytotoxicity in AML cells. To determine whether P-gp, MRP1 and BCRP affect the cytotoxic response to AVE9633 and DM4, different cell lines specifically expressing P-gp, MRP1 and BCRP were used. Our data demonstrate that MRP1 and BCRP did not affect AVE9633- and/or DM4-induced cytotoxicity in HL60/ADR and K562/BCRP cells, which respectively express MRP and BCRP, compared to the parental HL60 and K562 cells. The MRP and BCRP inhibitors, MK571 and FTC, failed to enhance cytotoxicity in these MRP- and BCRP-positive cells. We also showed that P-gp function attenuated AVE9633- and/or DM4-induced cytotoxicity in HL60/DNR, K562/HHT40, K562/HHT90 and K562/Dox cells, and that Zosuquidar restored their sensitivity. However, it seems that we cannot extrapolate these findings from AML cell lines to the clinical setting, because P-gp activity in HL60/DNR, K562/HHT40, K562/HHT90 and K562/DOX cells showed D values of 0.98 ± 0.004, 0.41 ± 0.01, 0.83 ± 0.05 and 0.99 ± 0.01 respectively, which are far higher than those observed in the AML patient cells. Furthermore, the plasma levels obtained in the AVE9633 clinical trials with doses ranging between 15 mg/m^2 ^and 260 mg/m^2 ^were 7–119 μg/ml (46 – 783 nM), which is much higher than the IC_50 _obtained in the cell lines.

Sensitivity to AVE9633 or DM4 was examined in samples of cells from 25 AML patients. The doses of AVE9633 or DM4 tested were from 1.5 nM to 500 nM (76 μg/ml), which is in line with the plasma concentration of 69 μg/ml observed in patients undergoing AVE9633 therapy at a dose of 150 mg/m^2 ^[6]. Among the AML patients tested, the cells from 10 (40%) were non-responsive to AVE9633 and/or DM4 and did not exhibit high P-gp activity; cells from only three patients had moderate P-gp activity (D>0.2, but D<0.3). Among 15 (60%) responsive cell samples, those from two patients showed high P-gp activity (D> 0.3), and those from four patients showed weak P-gp activity (D>0.2, but <0.3). These results suggest that P-gp activity does not play a pivotal role in chemoresistance to AVE9633 or DM4.

The patient cells that were resistant to AVE9633 were also resistant to DM4, and resistance was not related to CD33 expression. This suggests that other chemoresistance mechanisms are involved in AVE9633- or DM4-induced cytotoxicity. DM4, an antimitotic agent like other Vinca alkaloids, binds preferentially to α/β-tubulin heterodimers and inhibits tubulin polymerization and microtubule assembly, increasing microtubule destabilization, which leads to a potent suppression of microtubule dynamics and results in mitotic block and subsequent apoptotic cell death [7]. The balance between polymerized and non-polymerized tubulin is an important determinant of the response to Vinca alkaloid-based chemotherapy regimens in childhood ALL [22]. Clinical evidence shows that βIII-tubulin expression is involved in the resistance to taxanes and Vinca alkaloids in lung [23-25], breast [26,27] and ovarian cancers [28,29]. In addition, βIII-tubulin siRNA sensitizes cancer cells to tubulin-binding chemotherapeutic drugs [30]. This offers a rationale for investigating the involvement of different subtypes of tubulin in resistance to AVE9633/DM4, and for studying the benefit of AVE9633 in combination with the conventional anthracycline AML regimens, resistance to which is related to the expression of ABC proteins.

## Conclusion

P-gp activity, but not MRP1 and BCRP, attenuated AVE9633 and DM4 cytotoxicity in myeloid cell lines. Zosuquidar, a potent specific P-gp inhibitor, restored the sensitivity of those cells expressing P-gp to both AVE9633 and DM4. However, P-gp activity is not a crucial mechanism of chemoresistance to AVE9633 in AML patient cells, in contrast to the Gemtuzumab ozogamicin (GO) immunoconjugate. AVE9633 may be a potent cytotoxic chemotherapy for AML patients whose blasts express P-gp, especially older patients, and it may be beneficial in combination with the conventional anthracycline AML regimens, resistance to which is related to the expression of ABC proteins. Other mechanisms such as microtubule alteration could play an important role in chemoresistance to AVE9633.

## Competing interests

We received funding from Sanofi-Aventis for investigating the mechanism of chemoresistance to AVE9633 and DM4. Claudia Zuany-Amorim is an employee of Sanofi-Aventis.

## Authors' contributions

RT designed the study, the manuscript and some of the experiments. SC performed the major experiments. JYP and AMF participated in all flow cytometric analysis. CZA assisted with drug preparation and manuscript revision. ZM collected the AML patient blood. FF and EC participated in completing the clinical data. HM helped us with the BCRP study. OL and JPM participated in the design and manuscript revision. All authors read and approved the final manuscript.

## Pre-publication history

The pre-publication history for this paper can be accessed here:

http://www.biomedcentral.com/1471-2407/9/199/prepub

## Supplementary Material

Additional file 1 **Characteristics of patients' cells, P-gp activity, CD33 expression and sensitivity of AML cells to DM4, AVE9633 and GO in the presence or absence of the P-gp modulator Zosuquidar**. The data provided the characteristics of patients' cells and the sensitivity of patient cells to DM4, AVE9633 and GO. Click here for file

## References

[B1] Tallman MS New strategies for the treatment of acute myeloid leukemia including antibodies and other novel agents American Society of Hematology Education Program. Hematology 2005 1 143 150 10.1182/asheducation-2005.1.143 16304372

[B2] Stock W Controversies in Treatment of AML: Case-based Discussion American Society of Hematology Education Program. Hematology 2006 1 185 91 10.1182/asheducation-2006.1.185 17124059

[B3] Bross PF Beitz J Chen G Chen XH Duffy E Kieffer L Roy S Sridhara R Rahman A Williams G Pazdur R Approval summary: gemtuzumab ozogamicin in relapsed acute myeloid leukemia Clin Cancer Res 2001 7 1490 6 11410481

[B4] Pierelli L Teofili L Menichella G Rumi C Paoloni A Iovino S Puggioni PL Leone G Bizzi B Further investigations on the expression of HLA-DR, CD33 and CD13 surface antigens in purified bone marrow and peripheral blood CD34+ haematopoietic progenitor cells Br J Haematol 1993 84 24 30 10.1111/j.1365-2141.1993.tb03021.x 7687858

[B5] Dinndorf PA Andrews RG Benjamin D Ridgway D Wolff L Bernstein ID Expression of normal myeloid-associated antigens by acute leukemia cells Blood 1986 67 1048 53 2937468

[B6] Legrand Ollivier Vidriales Maria B Thomas Xavier Dumontet Charles Vekhoff Anne Morariu-Zamfir Rodica Lambert John San Miguel Jesus F Marie Jean-Pierre An Open Label, Dose Escalation Study of AVE9633 Administered as a Single Agent by Intravenous (IV) Infusion Weekly for 2 Weeks in 4-Week Cycle to Patients with Relapsed or Refractory CD33-Positive Acute Myeloid Leukemia (AML). 49th meeting of the American Society of Hematology Blood 2007 110

[B7] Remillard S Rebhun LI Howie GA Kupchan SM Antimitotic activity of the potent tumor inhibitor maytansine Science 1975 189 4207 1002 5 10.1126/science.1241159 1241159

[B8] Chabner BA Levine AS Johnson BL Young RC Initial clinical trials of maytansine, an antitumor plant alkaloid Cancer Treat Rep 1978 62 429 33 348311

[B9] Blum RH Kahlert T Maytansine: A phase I study of an ansa macrolide with antitumor activity Cancer Treat Rep 1978 62 435 438 348312

[B10] Cabanillas F Rodriguez V Hall SW Burgess MA Bodey GP Freireich EJ Phase I study of maytansine using a 3-day schedule Cancer Treat Rep 1978 62 425 8 348310

[B11] Eagan RT Ingle JN Rubin J Frytak S Moertel CG Early clinical study of an intermittent schedule for maytansine (NSC-153858): brief communication J Natl Cancer Inst 1978 60 93 6 628025 10.1093/jnci/60.1.93

[B12] Issell BF Crooke ST Maytansine Cancer Treat Rev 1978 5 199 207 10.1016/S0305-7372(78)80014-0 367597

[B13] Marie JP Legrand O MDR1/P-GP expression as a prognostic factor in acute leukemias Adv Exp Med Biol 1999 457 1 9 10500774 10.1007/978-1-4615-4811-9_1

[B14] Leith CP Kopecky KJ Godwin J McConnell T Slovak ML Chen IM Head DR Appelbaum FR Willman CL Acute myeloid leukemia in the elderly: assessment of multidrug resistance (MDR1) and cytogenetics distinguishes biologic subgroups with remarkably distinct responses to standard chemotherapy. A Southwest Oncology Group study Blood 1997 89 3323 9 9129038

[B15] Legrand O Perrot JY Simonin G Baudard M Marie JP JC-1: a very sensitive fluorescent probe to test P-gp activity in adult acute myeloid leukemia Blood 2001 97 502 8 10.1182/blood.V97.2.502 11154229

[B16] Benderra Z Faussat AM Sayada L Perrot JY Tang R Chaoui D Morjani H Marzac C Marie JP Legrand O MRP3, BCRP, and P-glycoprotein activities are prognostic factors in adult acute myeloid leukemia Clin Cancer Res 2005 11 7764 72 10.1158/1078-0432.CCR-04-1895 16278398

[B17] Walter RB Raden BW Hong TC Flowers DA Bernstein ID Linenberger ML Multidrug resistance protein attenuates gemtuzumab ozogamicin-induced cytotoxicity in acute myeloid leukemia cells Blood 2003 102 1466 73 10.1182/blood-2003-02-0396 12689934

[B18] Tang R Faussat AM Perrot JY Marjanovic Z Cohen S Storme T Morjani H Legrand O Marie JP Zosuquidar restores drug sensitivity in P-glycoprotein expressing acute myeloid leukaemia (AML) BMC Cancer 2008 8 51 18271955 10.1186/1471-2407-8-51 PMC2258302

[B19] Linenberger ML Hong T Flowers D Sievers EL Gooley TA Bennett JM Berger MS Leopold LH Appelbaum FR Bernstein ID Multidrug-resistance phenotype and clinical responses to gemtuzumab ozogamicin Blood 2001 98 988 94 10.1182/blood.V98.4.988 11493443

[B20] Zhou DC Ramond S Viguié F Faussat AM Zittoun R Marie J-P Progressive resistance to homoharringtonine in human myeloleukemia K562 cells: relationship to sequential emergence of MRP and MDR1 gene overexpression and MDR1 gene translation Int J Cancer 1996 65 365 71 10.1002/(SICI)1097-0215(19960126)65:3<365::AID-IJC15>3.0.CO;2-9 8575859

[B21] Yanase K Tsukahara S Asada S Ishikawa E Imai Y Sugimoto Y Gefitinib reverses breast cancer resistance protein-mediated drug resistance Mol Cancer Ther 2004 3 1119 25 15367706

[B22] Ong V Liem NL Schmid MA Verrills NM Papa RA Marshall GM Mackenzie KL Kavallaris M Lock RB A role for altered microtubule polymer levels in vincristine resistance of childhood acute lymphoblastic leukemia xenografts J Pharmacol Exp Ther 2008 324 434 42 10.1124/jpet.107.128926 17986648

[B23] Rosell R Scagliotti G Danenberg KD Lord RV Bepler G Novello S Cooc J Crinò L Sánchez JJ Taron M Boni C De Marinis F Tonato M Marangolo M Gozzelino F Di Costanzo F Rinaldi M Salonga D Stephens C Transcripts in pretreatment biopsies from a three-arm randomized trial in metastatic non-small-cell lung cancer Oncogene 2003 22 3548 53 10.1038/sj.onc.1206419 12789263

[B24] Dumontet C Isaac S Souquet PJ Bejui-Thivolet F Pacheco Y Peloux N Frankfurter A Luduena R Perol M Expression of class III beta tubulin in non-small cell lung cancer is correlated with resistance to taxane chemotherapy Bull Cancer 2005 92 E25 30 15749640

[B25] Sève P Lai R Ding K Winton T Butts C Mackey J Dumontet C Dabbagh L Aviel-Ronen S Seymour L Whitehead M Tsao MS Shepherd FA Reiman T Class III beta-tubulin expression and benefit from adjuvant cisplatin/vinorelbine chemotherapy in operable non-small cell lung cancer: analysis of NCIC JBR.10 Clin Cancer Res 2007 13 994 9 10.1158/1078-0432.CCR-06-1503 17289895

[B26] Tommasi S Mangia A Lacalamita R Bellizzi A Fedele V Chiriatti A Thomssen C Kendzierski N Latorre A Lorusso V Schittulli F Zito F Kavallaris M Paradiso A Cytoskeleton and paclitaxel sensitivity in breast cancer: the role of beta-tubulins Int J Cancer 2007 120 2078 85 10.1002/ijc.22557 17285590

[B27] Hasegawa S Miyoshi Y Egawa C Ishitobi M Taguchi T Tamaki Y Monden M Noguchi S Prediction of response to docetaxel by quantitative analysis of class I and III beta-tubulin isotype mRNA expression in human breast cancers Clin Cancer Res 2003 9 2992 7 12912947

[B28] Mozzetti S Ferlini C Concolino P Filippetti F Raspaglio G Prislei S Gallo D Martinelli E Ranelletti FO Ferrandina G Scambia G Class III beta-tubulin overexpression is a prominent mechanism of paclitaxel resistance in ovarian cancer patients Clin Cancer Res 2005 11 298 305 15671559

[B29] Ferrandina G Zannoni GF Martinelli E Paglia A Gallotta V Mozzetti S Scambia G Ferlini C Class III beta-tubulin overexpression is a marker of poor clinical outcome in advanced ovarian cancer patients Clin Cancer Res 2006 12 2774 9 10.1158/1078-0432.CCR-05-2715 16675570

[B30] Gan PP Pasquier E Kavallaris M Class III beta-tubulin mediates sensitivity to chemotherapeutic drugs in non small cell lung cancer Cancer Res 2007 67 9356 63 10.1158/0008-5472.CAN-07-0509 17909044

