# Supplementary material for: P-gp activity is a critical resistance factor against AVE9633 and DM4 cytotoxicity in leukaemia cell lines, but not a major mechanism of chemoresistance in cells from acute myeloid leukaemia patients
Source: BMC Cancer. 2009 Jun 23;9:199. doi: 10.1186/1471-2407-9-199 (PMC2708190; doi:10.1186/1471-2407-9-199)
Supplement: Additional file 1 — Characteristics of patients' cells, P-gp activity, CD33 expression and sensitivity of AML cells to DM4, AVE9633 and GO in the presence or absence of the P-gp modulator Zosuquidar. The data provided the characteristics of patients' cells and the sensitivity of patient cells to DM4, AVE9633 and GO. [file 1471-2407-9-199-S1.doc]

Additional file 1

**a. NT:** No tested due to missing the cells.
